# Patterns of contribution to citizen science biodiversity projects increase understanding of volunteers’ recording behaviour

**DOI:** 10.1038/srep33051

**Published:** 2016-09-13

**Authors:** Elizabeth H. Boakes, Gianfranco Gliozzo, Valentine Seymour, Martin Harvey, Chloë Smith, David B. Roy, Muki Haklay

**Affiliations:** 1 Centre for Biodiversity & Environment Research, Department of Genetics, Evolution & Environment, University College London, Gower Street, London WC1E 6BT, UK; 2 Department of Computer Science, University College London, Gower Street, London WC1E 6BT, UK; 3 Department of Civil, Environmental and Geomatic Engineering, University College London, Gower Street, London WC1E 6BT, UK; 4 Faculty of Science, The Open University, Walton Hall, Milton Keynes, MK7 6AA, UK; 5 Greenspace Information for Greater London CIC, Dean Bradley House, 52 Horseferry Road, London, SW1P 2AF; 6 Biological Records Centre, NERC Centre for Ecology and Hydrology Wallingford, Crowmarsh Gifford, Wallingford, Oxon, OX10 8BB, UK

## Abstract

The often opportunistic nature of biological recording via citizen science leads to taxonomic, spatial and temporal biases which add uncertainty to biodiversity estimates. However, such biases may also give valuable insight into volunteers’ recording behaviour. Using Greater London as a case-study we examined the composition of three citizen science datasets – from Greenspace Information for Greater London CIC, iSpot and iRecord - with respect to recorder contribution and spatial and taxonomic biases, i.e. when, where and what volunteers record. We found most volunteers contributed few records and were active for just one day. Each dataset had its own taxonomic and spatial signature suggesting that volunteers’ personal recording preferences may attract them towards particular schemes. There were also patterns across datasets: species’ abundance and ease of identification were positively associated with number of records, as was plant height. We found clear hotspots of recording activity, the 10 most popular sites containing open water. We note that biases are accrued as part of the recording process (e.g. species’ detectability) as well as from volunteer preferences. An increased understanding of volunteer behaviour gained from analysing the composition of records could thus enhance the fit between volunteers’ interests and the needs of scientific projects.

Public participation in scientific research, or ‘citizen science’, has become a well-established branch of biodiversity monitoring^1^,^2^ and has been used for multiple conservation science purposes, for example to estimate species trends, map species distributions and study climate change ecology^1^,^3^. However, the mix of opportunistic records and focussed surveys that tend to constitute citizen science data mean that data can contain a variety of biases^4^ and often may better represent distributions of recorders than species^5^. These biases are usually discussed in the literature as obstacles to be controlled for^6^,^7^,^8^ but such biases can also be viewed in a positive light because they provide valuable information regarding volunteers’ recording behaviour.

An increased understanding of volunteers’ recording activity patterns (i.e. when and how regularly they record) and their preference for recording particular taxa or at particular sites would allow recording schemes to tailor their projects to enhance the fit between science and volunteers’ enjoyment and thus presumably increase participation^9^,^10^. Modelling volunteer behaviour can also be used to predict taxa or sites within a project that are less likely to be surveyed by volunteers^11^.

Bias may arise through (i) uneven sampling over time, e.g. a spate of recording a taxa because of a particular project, (ii) uneven spatial coverage, e.g. volunteers recording more in areas they enjoy visiting or can easily access, (iii) uneven sampling effort per visit, e.g. volunteers recording or reporting only those species that are of interest to them or that they can identify, and (iv) uneven detectability across space and time, e.g. some taxa being easier to observe than others^8^. The implications of these biases are discussed more fully by Isaac and Pocock^4^.

It should be remembered that unlike most data, the number of biological records does not correspond directly to their information content^4^; the temporal and spatial coverages and field sampling method are all relevant to a dataset’s contribution to estimating the parameter of interest, e.g. change in species’ distributions over time. If the information content of citizen science datasets is to be increased we need to understand the information content in individual volunteers’ contributions. For example, what kinds of data can we expect from recruiting many volunteers who collect only a couple of records versus a few experienced volunteers who collect many records? By ‘profiling’ volunteers according to their activity patterns and analysing the information content of data from each profile group, citizen science projects can better allocate financial resources with respect to volunteer recruitment versus training.

Studies of people’s preferences for biodiversity have found that participants tend to prefer areas of higher species richness^12^,^13^ and higher taxonomic diversity^14^,^15^. However, volunteers will have individual reasons for participating in particular citizen science projects and, without investigating their data collection habits in detail, it is hard to predict the nature of spatial and taxonomic bias. For example some individuals will record from their gardens whilst others travel to more remote sites; some will show a preference for common species whilst others tend to seek rare species.

The concept of using the bias in citizen science data to better understand volunteer behaviour is relevant across projects internationally although the nature of such bias is likely to vary both between and within countries depending on the biota and landscape, traditions of participation, funding for schemes and access to natural sites. We examine and compare the composition of citizen science datasets held by one regional records centre and two national (UK) recording initiatives respectively: Greenspace Information for Greater London Community Interest Company (GiGL), iSpot and iRecord, using Greater London as a case study. These three volunteer-contributed datasets represent a broad range of contributors. GiGL (http://www.gigl.org.uk/) is London’s environmental records centre, collating wildlife observations via data sharing agreements with recorders and natural history organisations; iSpot (www.ispotnature.org) is a web application developed by the Open University to help anyone, from beginner to expert, with species identification via the submission of a photograph to an online social network; iRecord (www.brc.ac.uk/irecord) is an online data portal that allows observers to manage and share their wildlife records and it also stores data from partner organisations who agree to share data, for example, iRecord Ladybird (www.ladybird-survey.org). We explore contribution patterns within volunteer-collected data by a) profiling volunteers’ engagement patterns, b) mapping data collection sites and c) investigating the taxa that are more frequently recorded and their ecological traits.

## Results

The GiGL dataset contained 1,102,746 records from 7715 volunteers, iSpot 13,352 records from 887 volunteers and iRecord 9,505 records from 1210 volunteers.

### Engagement profile analysis

We found engagement metrics to cluster into three distinguishable groupings that we termed ‘dabbler’, ‘steady’ and ‘enthusiast’ (Table 1). These were shown to be the number that best optimised the trade-offs between the number of groups and the within group sum of squares. This was validated in Averaged Silhouette widths with scores above 0.51 indicating sufficient partitioning (iSpot 0.56; iRecord 0.58; GiGL 0.51). Engagement profiles present a gradient in engagement metrics. For example, those classified as ‘dabblers’ have the highest activity ratio (number of days a volunteer was active divided by the total number of days they were linked to the dataset), least amount of observations, highest variation in periodicity and lowest relative activity duration (the number of days a volunteer was active divided by the overall study period in days). By contrast, those classified as ‘enthusiasts’ tend to be those that are increasingly long term and persistent, dedicating more time and observations, yet account for the least number of participants. Those that have been classified as ‘steady’ volunteers have a profile type mid-way between the two other profiles. This is supported by significant Spearman’s rank correlations being observed between each of the engagement metrics (*p* < 0.001).

Dabblers make up the largest group of contributors with a gradual decline noticeable from steady through to enthusiasts (Table 1). Comparatively, iRecord presents a higher proportion of dabblers than those found in iSpot and GiGL although it also has the highest proportion of enthusiasts. 67–89% of the dabblers were active for one day only (GiGL 67%; iRecord 85%; iSpot 89%).

In line with the results of the engagement analysis, there was a repeated pattern across datasets and taxonomic groups of a few volunteers contributing many records and many volunteers contributing few records. For example GiGL’s four most productive recorders contributed over 10% of the >1 million GiGL records but at the other end of the scale, around 35% of GiGL recorders (2708) submitted only one record.

### Spatial analysis

**Figure 1:**
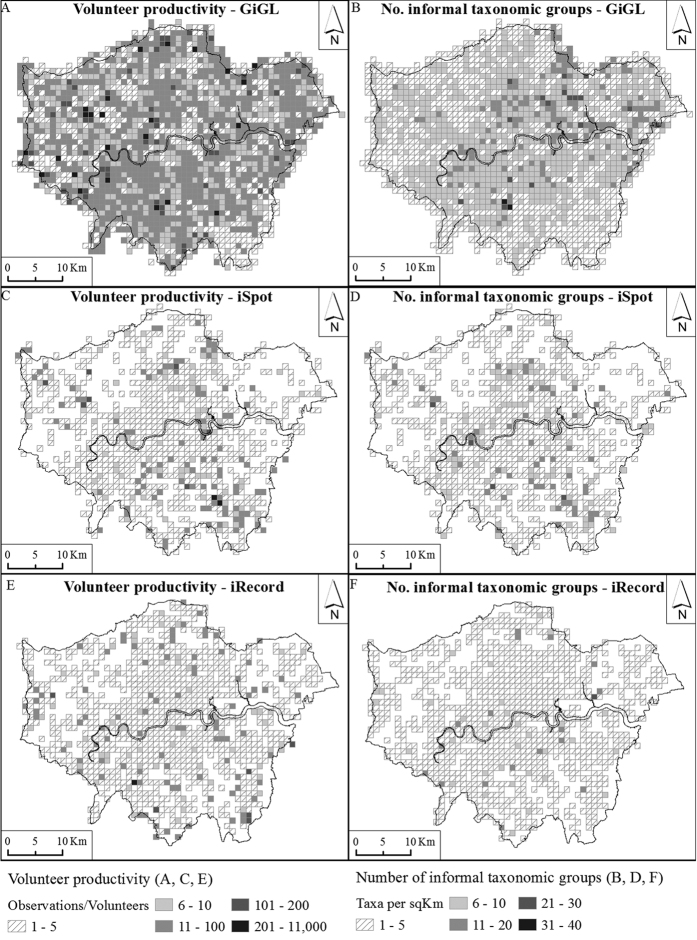
The spatial distribution of volunteer productivity and number of informal taxonomic groups recorded for (**A,B**) the GiGL, (**C,D**) iSpot and (**E,F**) iRecord datasets. Figure drawn using ESRI ArcGIS 10.1.1 (http://www.esri.com/software/arcgis), QGIS version 2.8.0-Wien (http://www.qgis.org/en/site/) and EDINA Digimap Ordnance Survey Service (http://digimap.edina.ac.uk). Contains OS data © Crown copyright and database right 2016. Additional data sourced from public sector information licensed under the Open Government Licence v1.0.

**Figure 2:**
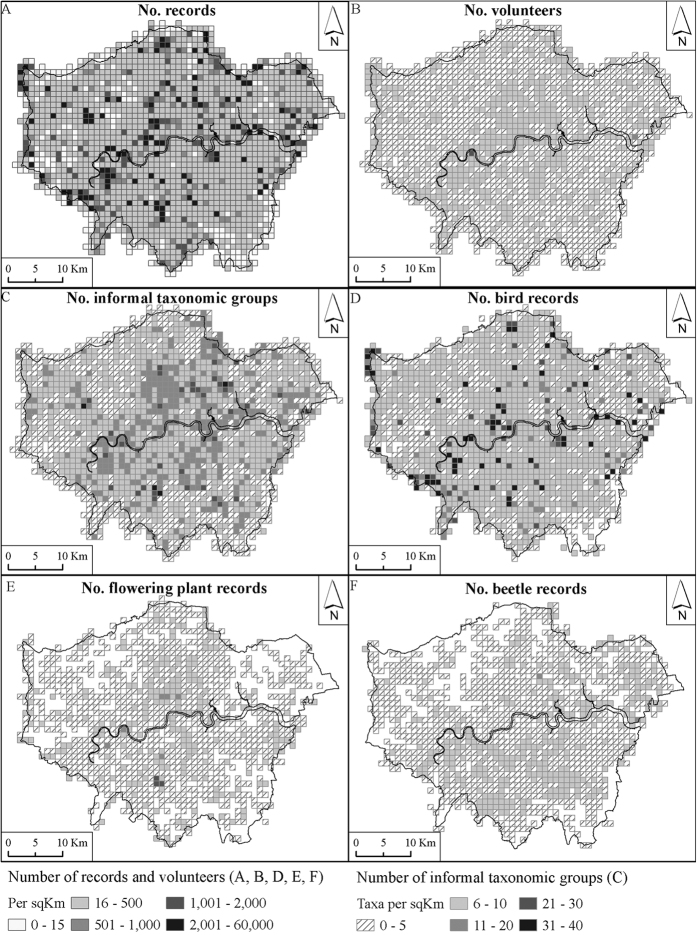
The spatial distribution of the total numbers of (**A**) records, (**B**) volunteers, (**C**) informal taxonomic groups recorded, (**D**) bird records, (**E**) flowering plant records and (**F**) beetle records. Figure drawn using ESRI ArcGIS 10.1.1 (http://www.esri.com/software/arcgis), QGIS version 2.8.0-Wien (http://www.qgis.org/en/site/) and EDINA Digimap Ordnance Survey Service (http://digimap.edina.ac.uk). Contains OS data © Crown copyright and database right 2016. Additional data sourced from public sector information licensed under the Open Government Licence v1.0.

The number of grid cells for which records had been submitted at a 1 km resolution or better was GiGL 1780; iSpot 899; iRecord 1071, corresponding to 91%, 46% and 55% respectively of the total 1950 cells in Greater London (Fig. 1). Despite having fewer total records than iSpot, iRecord’s volunteers covered a greater geographic area than iSpot’s. Figures 1 and 2 demonstrate the spatial variability of data collection activities across the area of Greater London.

Figure 1 shows the spatial distribution of volunteer productivity (mean number of records per volunteer) and number of informal taxonomic groups recorded (see Supplementary Table S1) for each dataset. It can be seen that the distribution of volunteer productivity and number of informal taxonomic groups recorded varies considerably across grid cells. However, the spatial distributions of volunteer productivity were significantly correlated between datasets (Spearman’s Rank correlation): GiGL and iSpot *ρ* (1778) = 0.235, *p* < 0.001; GiGL and iRecord: *ρ* (1778) = 0.271, *p* < 0.001; iSpot and iRecord: *ρ* (1069) = 0.333, *p* < 0.001, as were the number of informal taxonomic groupings recorded per grid cell; GiGL and iSpot *ρ* (1778) = 0.378, *p* < 0.001; GiGL and iRecord: *ρ* (1778) = 0.315, *p* < 0.001; iSpot and iRecord: *ρ* (1069) = 0.395, *p* < 0.001. The correlation coefficients are not particularly high but the results suggest that the same grid cells are broadly more/less productive for all datasets for both number of records per volunteer and number of informal taxonomic groups recorded.

Although the total number of records per grid cell summed across datasets (Fig. 2A) was not uniform across the study area the spatial distribution of records was significantly correlated between datasets (Spearman’s Rank correlation); GiGL and iSpot: *ρ* (1778) = 0.373, *p* < 0.001; GiGL and iRecord *ρ* (1778) = 0.402, *p* < 0.001; iSpot and iRecord: *ρ* (1069) = 0.544, *p* < 0.001. The total number of volunteers recording in each grid cell was also not uniform (Fig. 2B). The number of records per grid cell was highly correlated with the number of volunteers, *ρ* (1801) = 0.795, *p* < 0.001. The number of informal taxonomic groups recorded (Fig. 2C) was also correlated with the number of records per grid cell, *ρ* (1801) = 0.650, *p* < 0.001, the number of volunteers per grid cell, *ρ* (1801) = 0.665, *p* < 0.001 and volunteer productivity, *ρ* (1801) = 0.451, *p* < 0.001.

The grid cells containing the 10 highest numbers of total records, total volunteers and number of informal taxonomic groups are given in the Supplementary Table S2. Given the correlations of number of informal taxonomic groups with number of records and number of volunteers it is interesting that the cell in which the London Wetland Centre is sited contained both the highest number of records and the highest number of volunteers but did not feature in the top 10 cells for taxonomic groups recorded. We also noted that 9 of the 10 cells with the highest number of records and all of the 10 cells with the highest number of volunteers contained open water (‘bluespace’), suggesting that such sites are particularly popular with recorders.

The distributions of bird, beetle and flowering plant records (Fig. 2D–F) are significantly correlated (birds and flowering plants: *ρ* (1691) = 0.483, *p* < 0.001; birds and beetles: *ρ* (1695) = 0.433, *p* < 0.001; flowering plants and beetles: *ρ* (1479) = 0.379, *p* < 0.001) although the correlation coefficients are all <0.5 suggesting there is reasonable variation in where volunteers record particular taxa. The top 10 hotspots for total numbers of observations of the groups of birds, flowering plants and beetles are given in the Supplementary Table S3. There are only two sites which overlap all groups, the London Wetland Centre and Rainham Marshes Nature Reserve. It is interesting to note, however, that despite attracting many volunteers, the productivity, i.e. mean number of records per volunteer, in the grid cell containing the London Wetland Centre was comparatively low.

### Taxonomic analysis

**Figure 3:**
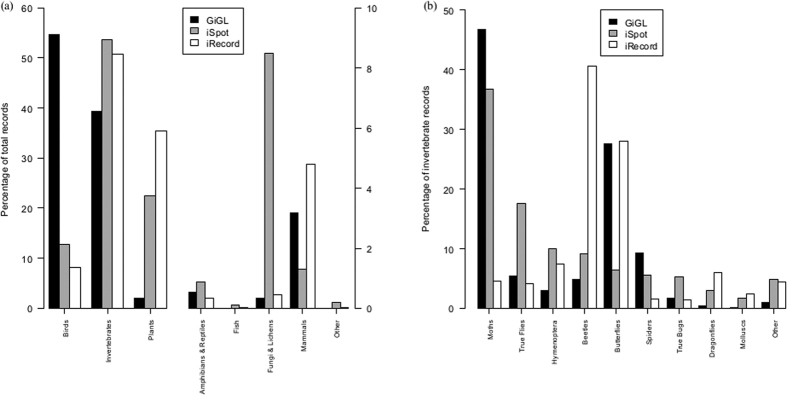
The percentage of records (**a**) per higher taxonomic group and (**b**) per invertebrate group for the GiGL, iSpot and iRecord data sets.

It can be seen that records are biased towards particular higher taxonomic groups but that these biases differ between datasets (Fig. 3a). (The ‘higher’ taxonomic groups as used by the UK recording community are birds, invertebrates, plants, amphibians & reptiles, fish, fungi & lichens, mammals and others). For example, birds make up nearly 55% of GiGL records but only 12% and 8% of iSpot and iRecord records, respectively. Conversely, plants receive much more coverage by iSpot and iRecord (23% and 36% respectively) than by GiGL (2%).

Invertebrates comprise a large part of each dataset (40–54%) but subgroupings using the recording community’s ‘informal’ taxonomic groupings (Supplementary Table S1) again show differences between datasets (Fig. 3b). For example, within Greater London, moths receive little representation in iRecord (4% of invertebrate records) compared to iSpot (37%) and GiGL (47%) with beetle records being iRecord’s largest invertebrate group (41%).

The number of recorders per taxonomic group did not follow a consistent pattern across datasets (Table 2). In general, GiGL had considerably more recorders per group, reflecting its far greater number of records and longer duration. However, there are some discrepancies. iRecord’s beetle recorders are not all that fewer than GiGL’s, the datasets have a similar number of dragonfly recorders and iRecord has more true bug recorders. iSpot has considerably more fungus recorders than the other two datasets and more true bug recorders.

In all taxonomic groups, GiGL has a greater mean number of records per recorder (Table 2). There is a particularly high mean number of records per GiGL moth recorder, presumably reflecting the large number of moths attracted to light traps (see Discussion). Despite similar numbers of beetle and dragonfly recorders across GiGL and iRecord, GiGL recorders are, on average, recording 10 and 6 times as many records respectively.

In all taxonomic groups except for dragonflies, GiGL covers more species than the other two datasets (Table 2), as would be expected from its greater number of records and longer history. GiGL is not restricted to on-line methods of data capture and may thus attract a wider range of volunteers which may also increase species coverage. Despite the greater number of iSpot’s fungus and true bug recorders, its species coverages are much lower than GiGL’s for these groups.

Number of records per bird species was significantly correlated with species’ identification difficulty, there being fewer records of species that are harder to identify within all datasets: GiGL, *F* (1,367) = 131.6, *p* < 0.001; iSpot, *F* (1,167) = 32.5, *p* < 0.001; iRecord *F* (1,78) = 5.07, *p* = 0.03). Native bird species received higher numbers of records than non-native for GiGL, *F* (1,399) = 92.8, *p* < 0.001, and iSpot, *F* (1,174) = 6.9, p = 0.009 but there was no significant relationship between number of records of native and non-native birds in iRecord. Common bird species were more heavily recorded in all datasets: GiGL, *F* (1,321) = 317.5 *p* < 0.001; iSpot, *F* (1,150) = 43.6, *p* < 0.001; iRecord, *F* (1,77) = 4.5, *p* = 0.04. Green conservation status bird species (species with the lowest conservation priority) were more recorded than amber (higher conservation priority) or red status (highest conservation priority) although the relationship was not quite significant at the 0.05 level for iRecord: GiGL, *F* (1,227) = 10.4, *p* < 0.001; iSpot, *F* (1,126) = 6.7, *p* < 0.001; iRecord, *F* (1,71) = 2.9, *p* = 0.06. Non-migrant species had significantly more records than migrants for GiGL, *F* (1,238) = 55.3, *p* < 0.001, and iSpot, *F* (1,155) = 30.5, *p* < 0.001, but there was no significant difference within the iRecord bird records. Aquatic bird species were less recorded within iRecord than non aquatic species, *F* (1,80) = 7.9, *p* = 0.006 but there was no significant difference for GiGL or iSpot. No significant relationships were found between number of bird records and species body length or species colour in any dataset. If the 8 tests are interpreted using a Bonferonni adjusted alpha level of 0.006, the iRecord data show no significant relationships other than aquatic birds are less recorded.

Number of records per flowering plant species was also significantly correlated with species’ identification difficulty, there being fewer records of species that are harder to identify: GiGL, *F* (1,1120) = 25.5, *p* < 0.001; iSpot, *F* (1,677) = 208.0, *p* < 0.001; iRecord, *F* (1,352) = 52.9, *p* < 0.001. The number of records per flowering plant species showed a positive relationship with species height within all datasets: GiGL, *F* (1,858) = 7.8, *p* = 0.005; iSpot, *F* (1,575) = 12.2, *p* < 0.001; iRecord, *F* (1,339) = 3.8, *p* = 0.05. More widely distributed species, as measured by the number of filled GB cells, were also more recorded within all datasets: GiGL, *F* (1,858) = 71.4, *p* < 0.001; iSpot, *F* (1,575) = 100.3, *p* < 0.001; iRecord, *F* (1,339) = 36.0, *p* < 0.001. There was no significant relationship in any of the datasets between number of records and species’ native status. If the 4 tests are interpreted using a Bonferroni adjusted alpha level of 0.012, the relationship between iRecord’s flowering plant records and species height would not be significant.

## Discussion

In common with other data compilations based largely on volunteer wildlife recording^16^,^17^ we have found a strong skew in participation, with a small number of recorders submitting many records. This pattern was repeated across datasets, the engagement profile analysis showing three distinct clusters of recorders, the greatest proportion being ‘dabblers’. The division of volunteers across profile types was similar for GiGL and iSpot but iRecord had a greater proportion of dabblers at the expense of its steady volunteers (Table 1), perhaps partly as a result of its more recent set-up and partly because of many of its records coming from the Ladybird App (www.ladybird-survey.org) which is aimed at the broader general public. High media profile citizen science projects like this may have an initial high uptake of ‘dabblers’ but also a rapid drop out rate^10^,^18^. Indeed, the ‘single-record’ volunteer type accounted for a considerable proportion of the volunteer abundance and is likely to be of importance for future research despite often being overlooked^18^, particularly as new shifts in volunteer patterns from the classic typology of habitual and long-term involvement to those more episodic, non-committal and self-oriented are emerging^19^. The recent technological developments that have increased ease of recording and identifying wildlife^20^ are at least partly responsible for the growth of biological recording in recent decades^4^. The question for citizen science programmes now is perhaps not how to attract volunteers but how to retain them^18^. Encouraging existing volunteers to further develop their skills would almost certainly increase species coverage and provide support for the next cohort of new volunteers. It is interesting that although iRecord had the highest proportion of dabblers, it also had the highest proportion of enthusiasts. This likely reflects iRecord’s breadth of appeal ranging from public-facing, high profile mobile apps, e.g. the Ladybird App, to tailored input systems suited to expert naturalists wanting to record across taxon groups (e.g. Pan-species Listing (www.brc.ac.uk/psl)).

The study demonstrates that the use of exploratory clustering techniques, as described by Ponciano and Brasileiro^21^, were applicable to other citizen science projects in an effort to explain volunteer’s engagement profiles. This is particularly useful for making comparative assessments between different volunteer programmes or citizen science initiatives that could be used to develop a universally standardised index or indices to monitor volunteer engagement. As this study showed, there is also the potential for alternative measures of volunteer’s engagement (e.g. number of species observations) to be incorporated into future assessments. One difficulty that we encountered was that the on-line recording mechanism of iSpot and iRecord meant that individual volunteers had unique recording IDs (although this does not preclude the possibility of individuals registering multiple times) but recorder IDs were not so clear in the GiGL dataset where recorder names such as Person, A. Person and Albert Person, suggest that a single recorder is often entered under multiple synonyms. Observations were also made by groups of people under one collective identity, e.g. the Wren Group, and the profiling analysis presented here excluded observations made by obviously identifiable groups. Further work is needed to understand whether the proportions of profiles are attributable to a specific volunteer programme, recording mechanisms or other forms of organisation. In addition, it might also be interesting to understand whether such volunteer profiles changed over time both longitudinally as well as at an individual scale^22^. The exploratory clustering technique does not allow one to extract volunteer identities from the volunteer profiles meaning we could not investigate potential differences in the species coverage of dabblers versus enthusiasts, for example. Such information would be extremely pertinent in allocating project resources to volunteer recruitment versus retainment/training.

Moving to the spatial distribution of observations, we found clear recording hotspots. Hotspots may be attributable to sites particularly rich in biodiversity, sites easily accessible to volunteers, sites which organise surveys with members of the public, sites regularly visited by recording societies, or to the influence of outstanding recorders (one cell had >30000 records from a single recorder). The London Wetland Centre and Rainham Marshes Nature Reserve both featured in the ten most recorded grid cells for birds, flowering plants and beetles. Both have high species diversity, observation hides and visitor amenities and thus are likely to attract volunteers with a wide range of interests and experience. Whilst there was spatial correlation of record numbers between bird, flowering plant and beetle records, the top 10 most recorded grid cells for each group also revealed taxon specific hotspots (see Supplementary Table S3). Birders seem to favour sites with open water, perhaps because of higher visibility and species richness.

Data were spatially correlated across datasets, the same cells being broadly more/less productive in terms of number of records, number of records per volunteer and number of informal taxonomic groups recorded, the strongest correlations being between iSpot and iRecord. Given the far greater number of GiGL recorders, however, it is not surprising that the correlations between GiGL and the other two datasets were lower. Further research could compare the distribution of habitats in which observations were made across the datasets and might increase insight into volunteers’ reasons for recording in particular areas. This finer-grain study would require records to be georeferenced at a resolution at least an order of magnitude higher than the majority of records in our study – many records stem from distribution atlases, for example, which record at the tetrad or monad level. The increase of data capture via smartphones with GPS chipsets could lead to a more precise way to record spatial information providing users are aware that records must be entered at the observation site, GPS precision is recorded and internet connectivity is available^20^.

The proportion of records in each higher taxonomic group differed considerably across datasets as did the 10 most recorded species of birds, flowering plants and beetles (Supplementary Table S4). Many factors will have influenced the number of records being contributed for the various taxa represented in the three sets of data analysed here. For example, some records will have been submitted as part of widely promoted citizen science projects, such as the Ladybird Recording Scheme and monitoring of the non-native Harlequin Ladybird (*Harmonia axyridis*)^23^,^24^. Some will result from the activities of organised recording groups and societies, such as the London Natural History Society (LNHS) (www.lnhs.org.uk) who contribute to GiGL or the British Dragonfly Society which contributes to iRecord. Others will be because of individuals’ interests.

If an individual recorder chooses to make records of a particular taxon, they will be encouraged to send their records to one of the recording schemes or the local records centre that can make use of them^25^. However, there are many different preferences among recording schemes for how they wish to receive data, with some promoting online systems such as iRecord, while others prefer to receive spreadsheets or emails. Volunteers will also have their own individual preferences for collecting data via smartphone or notebooks, for example, which may influence where they submit. Some taxon groups collect records via a network of ‘county recorders’ while others have a national recorder. Local environmental records centres collate data for all taxa for their particular geographical area. Thus a given individual recorder may choose, or be steered, to provide data to different collating systems depending on what taxa they record in which location. Of the three datasets, GiGL has a much longer history and many of the contributing volunteers have long lasting relationships with natural history societies and recording groups who share their records with GiGL, such as the LNHS.

iSpot volunteers have a different motivation from recording societies’ – observations are posted in order that the observer can identify a species. iSpot records thus have two key differences to those in GiGL and iRecord: (i) the observer does not need to be able to identify the species, (ii) the observer needs to supply a photograph of the species. The former removes bias caused by knowledge barriers that might arise in GiGL or iRecord but the latter imposes bias that will not be present in the other two datasets – some species are easier to photograph, and thus record, than others, for example a bird on open water versus one singing inside a hedge. However, there is no evidence to suggest that these two criteria have influenced data collection. As discussed in the next paragraph, identification difficulty of both bird and plant species was positively associated with number of records for the iSpot data and no association was found between aquatic birds and number of records.

At the species’ level, our analysis of traits of birds and flowering plants showed that species which were easier to identify were more likely to be recorded. Species of birds which were common, had green (low) conservation status, and were non-migratory were also more recorded, as were widely distributed species of plants, presumably because they were all encountered more frequently. A deeper insight into the value placed upon rarity or migratory species would have to control for abundance, perhaps comparing observed numbers of records to those expected using systematic surveys. Previous studies have suggested people place value on rarity^26^,^27^,^28^ but it is also possible that familiarity adds cultural value.

To what extent an individual species record is a measure of the recorders’ preference for that species is open to question. Arguably, the recorder’s choice is more meaningfully made at the higher taxon level (birds, moths, etc.). The individual species recorded from that group will depend on what species are available and most readily apparent in a given area, as well as a recorder’s knowledge, and is less likely to reflect active choice. Species vary greatly in their availability and apparency to recorders and different taxonomic groups tend to produce larger or smaller numbers of records. For example, a recorder may record thousands of individual moths representing over 100 species in the course of a year by using a light trap^29^, while a recorder specialising in beetles will have to work a lot harder to record similar numbers. In some cases, interest in particular taxa may be linked to high profile surveys or media coverage of invasive species^30^. Two invasives, the Harlequin ladybird (*Harmonia axyridis*) and Japanese knotweed (*Fallopia japonica*), for example, top iRecord’s most recorded beetle and flowering plant species (Supplementary Table S4). Whether such interest is sustained and the knowledge is assimilated and retained by the volunteer body or wanes could be revealed by a temporal analysis of recording habits. An understanding of the recording process is thus essential when interpreting bias with respect to volunteers’ behaviour. Biases due to the recording process, whether through the organisation of natural history societies, media coverage, species ‘recordability’ etc, must be separated from volunteer’s natural preferences for particular taxa and places. A short, publicly shared volunteer profile linked to a recorder’s ID and containing simple information such as approximate age, taxonomic interests and level of expertise in species identification would allow more in depth analysis of volunteer preferences and presumably would be useful for the individual citizen science projects themselves with regard to future volunteer recruitment.

By examining three citizen science datasets collected across Greater London we have shown that (i) whilst the different datasets have their own individual biases, the vast majority of volunteers contribute only a few records, (ii) recording hotspots tend to be taxon dependent although two sites (both with good visitor amenities) were popular across all three taxonomic groups investigated and (iii) species which are abundant or easily identified tend to be most recorded. Our approach allowed us to assess the recording preferences of ~10,000 volunteers and offers a relatively quick, albeit less detailed, alternative to surveys to gain insight into recording habits. A knowledge of what and where volunteers record not only helps us to understand how citizen science can increase its contribution to biodiversity monitoring but increases our understanding of how biodiversity is important to people. Immutable recorder IDs and records georeferenced to a fine resolution were essential for the analysis and should be strongly encouraged in all future recording initiatives and we suggest the introduction of recorder profiles to allow greater depth of analysis. Our study opens future questions about the recording process and behaviour of volunteers.

## Methods

### Data

UK biodiversity is exceptionally well recorded, representing almost one-eighth of the records on the Global Biodiversity Information Facility (GBIF)^3^. We used Greater London as a case-study. The administrative unit covers an area of 1,569 km^2^ and has ~9 million inhabitants. 33% is vegetated greenspace with a further 14% estimated to be private vegetated garden green space; 2.5% is bluespace such as rivers, canals and reservoirs. Greater London contains 185 protected wildlife sites with 19% of its area designated as Sites of Importance for Nature Conservation^31^.

We compared three volunteer-contributed datasets from Greenspace information for Greater London CIC (GiGL), iSpot and iRecord, encompassing a broad range of volunteer motivations and interests^32^,^33^,^34^.

GiGL (http://www.gigl.org.uk/) is London’s environmental records centre. It collates wildlife observations via data sharing agreements with recorders and natural history organisations. GiGL hosts online forms to facilitate partners’ data collection and a live form to collect species records that do not form part of any particular project (http://www.gigl.org.uk/submit-records/) although <0.1% of the GiGL data analysed here came from this latter source. GiGL’s collated dataset was begun in 1996 and although it also includes historic records, 99% of data are from 1975 onwards. Records are contributed not just by volunteers but by professional bodies such as the Royal Parks. Only surveys for which we were confident all records were collected in a voluntary capacity were included in this analysis. (This may include volunteers who are ‘professionals’ in their day-job but are contributing their own personal data collected in a voluntary capacity on their day-off.) The majority of GiGL’s volunteers are affiliated with natural history groups (e.g. The London Bat Group, Barn Hill Conservation Group) who will often be targeting a particular taxon or local area.

iSpot (www.ispotnature.org) is a web application developed by the Open University to help anyone, from beginner to expert, with species identification. Participants began contributing observations in mid-2009. Participants submit photographs to an online social network, purpose-designed to crowdsource the identification of organisms. Over 94% of observations submitted to iSpot receive a determination, verified at around 92% accuracy^33^. Users do not need to be able to identify the species they have photographed although they are encouraged to suggest an identification. Other iSpot users will then confirm or correct the suggested identification.

iRecord (www.brc.ac.uk/irecord) is an online data portal that allows observers to manage and share their wildlife records and thus is particularly useful for keener recorders and Panlisters (who record across all taxa). It started collecting observations in mid-2012. Sightings are checked by automated rules and by experts. Users can submit wildlife sightings via a general form or forms tailored to particular projects or taxonomic groups. iRecord has links with many established national taxon recording schemes and regional co-ordinating Local Record Centres and is helping those communities move on-line. iRecord stores data from partner organisations who agree to share data, some of which may be collating data for specific goals or using specific methodologies, for example, the Garden Bioblitz (www.gardenbioblitz.org) an annual survey in which volunteers list the wildlife in their garden, or iRecord Ladybird (www.ladybird-survey.org) which has been widely publicised by the media. iRecord contains a subset of data from iSpot for verification purposes and so this was removed to avoid duplication.

### Statistical analysis

All statistical analyses were performed in R version 3.1.0^35^. Due to the size of the GiGL dataset (over 1 million records), we examined only the ~88,000 records which could be assigned to an exact day and were recorded to a precision of 1 km or greater.

### Engagement profile analysis

Citizen science data exhibit participation inequality^36^ and the time, number of records and frequency of contribution can vary by several orders of magnitude between volunteers^21^. We identified volunteer engagement profiles broadly following the engagement metrics and clustering algorithms of Ponciano and Brasileiro^21^ but including volunteers who have been active for only a day because we wanted to profile our entire volunteer set. Anonymous records and records assigned to groups of people under one collective identity (where identifiable) were excluded from the analysis as we were looking at individual contributors’ patterns.

We used three of the four engagement characteristics defined by Ponciano and Brasileiro^21^: Activity Ratio, Relative Activity Duration and Variation in Periodicity. Activity Ratio is the number of days a volunteer was active divided by the total days they are linked to the dataset, i.e. all of the days between the first and last days on which they made observations. A volunteer was considered to be active if they made at least one observation on a single day. Relative Activity Duration is the number of days a volunteer was active divided by the overall study observation period in days. Variation in Periodicity is the average number of days elapsed between two sequential active days of an individual divided by the total average of days elapsed between active days of all individuals. Finally, the total number of species observations made by each volunteer was included as a further engagement metric, which informs us about each volunteer’s extent of engagement. We then normalised those engagement metrics to span 0 to 1.

Following this, we applied hierarchical cluster analysis (using the R package ‘cluster’^37^) using Ward’s Minimum Variance method to estimate the observed similarities and dissimilarities between volunteer’s engagement metrics. This enabled the datasets to be organised into distinguishable grouped clusters where no predefined number had been selected^38^. Drawing on this, we plotted the within group sum of squares by the number of clusters for each survey dataset to identify the number of grouped clusters. We applied the K-Means clustering approach to partition data points into the *k* number of groupings selected, which sorted data values according to the nearest mean at each cluster’s centre^39^. We then used an Averaged Silhouette Width (using pamk in the R package ‘fpc’^40^) to validate the numbers of clusters selected and evaluate each cluster’s degree of tightness and separation^41^. We used scores ≥0.51 as a reference to indicate sufficient partitioning. We used Spearman’s rank correlation coefficients to see whether relationships could be identified between each of the engagement metrics that would further explain volunteer engagement profiles.

### Spatial analysis

For the spatial exploration, only records with a precision of 1 km or greater were used. Records were aggregated to the 1 square km level of the British National Grid (BNG). For each dataset we tried to encapsulate the variety of reasons a grid cell might be a recording hotspot, e.g. the ‘home patch’ of one prolific volunteer, popular visitor spot, high taxonomic diversity, high species richness, by calculating the number of observations per grid cell, the number of volunteers that had recorded in each grid cell, the number of informal taxonomic groups recorded in each grid cell (see Supplementary Table S1 for a full list), the volunteer productivity per grid cell (defined as total number of observations/total number of volunteers) and the number of observations of birds, flowering plants and beetles per grid cell. Correlations of the aforementioned metrics across datasets were assessed using Spearman’s Rank tests. Correlations of number of observations and number of volunteers, number of observations and informal taxonomic groups recorded, number of volunteers and taxonomic richness, and volunteer productivity and number of informal taxonomic groups recorded were similarly assessed. The groups of birds, flowering plants and beetles were chosen to compare because they are popular with volunteers and accessible to beginners yet cross a diversity of taxa and volunteer interests - birders, for example, may record from different areas to botanists.

### Taxonomic analysis

To differentiate between taxa popular with highly enthusiastic individuals and taxa universally popular, the total number of records, number of volunteers, mean number of records per volunteer and number of species for each higher and informal taxonomic group was calculated. Informal taxonomic groupings are given in the Supplementary Table S1. Figures for the total number of species on the British list for each informal taxonomic group were taken from the MapMate database species dictionary (www.mapmate.co.uk) for all groups except fungi, for which the total was taken from the British Mycological Society’s online checklist (www.fieldmycology.net/GBCHKLST/gbchklst.asp).

We conducted a preliminary analysis into why some species are recorded more than others by investigating species traits of birds and flowering plants which might influence their appeal to volunteers and could be easily scored. Since the analysis was exploratory we did not apply a Bonferroni correction. Species traits for birds were identification difficulty, brightly coloured versus drab, log body length, rarity, UK conservation status, seasonal status and aquatic. Identification difficulty was scored numerically from 1–4 with 1 being the easiest to identify, as defined by the NBN Record Cleaner^42^, unlisted species were excluded. Birds were defined as brightly coloured if their plumage contained an easily distinguishable patch of blue, green, red or yellow. Body length for native species was taken from the British Trust for Onithology’s BirdFacts (http://www.bto.org/about-birds/birdfacts) and non-natives from miscellaneous sources. Rarity was scored as Common or Uncommon using the BirdGuides rarity levels (http://www.birdguides.com/species/) of ‘common’ for Common and ‘local’, ‘scarce, ‘rare’ and ‘mega’ for Uncommon; unlisted species were excluded. UK conservation status was scored as Green, Amber or Red, following the Birds of Conservation Concern 3 list^43^, non-natives were excluded. Migratory status of Migrant or Non-migrant was taken from the RSPB bird guide (http://www.rspb.org.uk/discoverandenjoynature/discoverandlearn/birdguide), non-natives were excluded. Birds not listed by the RSPB bird guide were scored as non-natives. Species were scored as aquatic if the species lived on or around water. Species traits for flowering plants were identification difficulty (NBN Record Cleaner^42^), native status, log height and number of 10 km squares the species occurs in in Britain as taken from PLANTATT^44^. Rarity was not used because almost all species were scored as common. For aquatic species without height data, length was substituted. Species not listed in the NBN Record Cleaner or PLANTATT (predominantly non-native species) were excluded from the respective analyses. We analysed these data using a generalised linear model with a quasipoisson distribution to correct for over-dispersion and number of records of a species as the dependent variable. An F-test was used to measure significance. Analyses were run independently on each dataset.

## Additional Information

**How to cite this article**: Boakes, E. H. *et al*. Patterns of contribution to citizen science biodiversity projects increase understanding of volunteers’ recording behaviour. *Sci. Rep.* **6**, 33051; doi: 10.1038/srep33051 (2016).

## Supplementary Material

Supplementary Information

## Figures and Tables

**Table 1:** A comparison of the volunteer engagement profiles across datasets.

**Profile**	**Dataset**	**No. observations**^a^	**Activity ratio^a^**[Fn t1-fn1]	**Relative activity durationa**[Fn t1-fn1]	**Variation in periodicity^a^**[Fn t1-fn1]	**No. volunteers**	**% of volunteers^b^**[Fn t1-fn2]
Dabbler	GiGL	33.00	0.60	0.030	0.78	470	67%
	iSpot	7.74	0.64	0.007	0.50	593	67%
	iRecord	6.13	0.62	0.014	1.01	969	84%
Steady	GiGL	48.00	0.50	0.050	1.08	223	32%
	iSpot	14.21	0.69	0.018	0.67	271	30%
	iRecord	264.5	0.23	0.059	0.29	132	11%
Enthusiast	GiGL	1524	0.20	0.220	4.99	9	1%
	iSpot	41.06	0.58	0.030	2.42	23	3%
	iRecord	1434	0.02	0.518	0.42	51	4%

^a^Values are given by the mean centroids from the cluster analyses.

^b^Values are rounded to the nearest whole number.

**Table 2:** A comparison of recording statistics across ten of the most recorded informal taxonomic groups.

Taxonomic group	**No. recorders**			**No. records/recorder**			**British species coverage**^a^		
	GiGL	iSpot	iRecord	GiGL	iSpot	iRecord	GiGL	iSpot	iRecord
Terrestrial mammal	534	73	142	65.7	2.3	3.2	72%(46)	36%(23)	44%(28)
Bird	5705	271	76	105.6	6.2	10.1	68%(399)	30%(176)	14%(82)
Beetle	608	164	539	34.4	10.2	3.6	28%(1142)	2%(134)	1%(56)
Dragonfly	122	73	110	13.3	2.9	2.6	46%(27)	51%(30)	31%(18)
Butterfly	623	103	135	191.7	4.4	10	72%(46)	58%(37)	52%(33)
Moth	464	231	35	437.3	11.2	6.2	63%(1452)	4%(94)	26%(604)
True bug	73	84	28	100.6	4.4	2.4	85%(513)	14%(84)	4%(27)
Spider	544	128	36	73.5	3.1	2.1	54%(372)	4%(26)	9%(64)
Fungus	68	11	12	52.2	5.7	1.7	6%(811)	0.1%(17)	2%(245)
Flowering plant	434	278	193	49.3	10.2	17.2	23%(1122)	8%(397)	15%(744)

^a^Number of species was taken from the British list (www.mapmate.co.uk and www.fieldmycology.net/GBCHKLST/gbchklst.asp) and is given in brackets.
