# Supplementary material for: Patterns of contribution to citizen science biodiversity projects increase understanding of volunteers’ recording behaviour
Source: Sci Rep. 2016 Sep 13;6:33051. doi: 10.1038/srep33051 (PMC5020317; doi:10.1038/srep33051)
Supplement: Supplementary Information [file srep33051-s1.pdf]

Supplementary Information

Patterns of contribution to citizen science biodiversity projects increase understanding of volunteers' recording behaviour.

Authors:

[*]Elizabeth H. Boakes[a], Gianfranco Gliozzo[b], Valentine Seymour[c], Martin Harvey[d], Chloë Smith[e], David B. Roy[f] and Muki Haklay[c]

[a]Centre for Biodiversity & Environment Research, Department of Genetics, Evolution & Environment, University College London, Gower Street, London WC1E 6BT, UK, e.boakes@ucl.ac.uk

[b]Department of Computer Science, University College London, Gower Street, London WC1E 6BT, UK, g.gliozzo@ucl.ac.uk

[c]Department of Civil, Environmental and Geomatic Engineering, University College London, Gower Street, London WC1E 6BT, UK, valentine.seymour.12@ucl.ac.uk; m.haklay@ucl.ac.uk

[d]Faculty of Science, The Open University, Walton Hall, Milton Keynes, MK7 6AA, UK, marharv@ceh.ac.uk

[e]Greenspace Information for Greater London CIC, Dean Bradley House, 52 Horseferry Road, London, SW1P 2AF, chloe.smith@gigl.org.uk

[f]Biological Records Centre, NERC Centre for Ecology and Hydrology Wallingford, Crowmarsh Gifford, Wallingford, Oxon, OX10 8BB, UK, dbr@ceh.ac.uk

Corresponding author: Elizabeth Boakes, e.boakes@ucl.ac.uk, +44 (0)776 202 1139

S1. The informal taxonomic groupings.

Acarine (Acari)

Alga

Amphibian

Annelid

Bird

Bony fish (Actinopterygii)

Bryozoan

Centipede

Chromist

Clubmoss

Colenterate (=cnidarian)

Conifer

Crustacean

False scorpion (Pseudoscorpiones)

Fern

Flatworm (Turbellaria)

Flowering plant

Fungus

Ginkgo

Harvestman (Opiliones)

Horsetail

Insect – alderfly (Megaloptera)

Insect – beetle (Coleoptera)

Insect – booklouse (Psocoptera)

Insect – butterfly

Insect – caddis fly (Trichoptera)

Insect – cockroach (Dictoptera)

Insect – dragonfly (Odonata)

Insect – earwig (Dermaptera)

Insect – hymenopteran

Insect – lacewing (Neuroptera)

Insect – mayfly (Ephemeroptera)

Insect – moth

Insect – orthopteran

Insect – scorpion fly (Mecoptera)

Insect – silverfish (Thysanura)

Insect – snakefly (Raphidioptera)

Insect – true bug (Hemiptera)

Insect – true fly (Diptera)

Jawless fish (Agnatha)

Lichen

Liverwort

Marine mammal

Millipede

Mollusc

Moss

Reptile

Roundworm (Nematoda)

Slime mould

Spider (Araneae)

Springtail (Collembola)

Stonewort

Symphylan

Terrestrial mammal

Two-tailed bristletail (Diplura)

S2. The ten grid cells with the highest number of records, number of volunteers, and taxonomic richness.

Observations		Volunteers		Taxonomic Richness	
TQ2277	The London Wetland Centre	TQ2277	The London Wetland Centre	TQ2967	Mitcham Common
TQ2596	High Barnet	TQ5280	Rainham Marshes Nature Reserve	TQ2868	Mitcham Common
TQ5280	Rainham Marshes Nature Reserve	TQ0493	Stocker's Lake	TQ4187	Wanstead Park
TQ1884	Alperton (Grand Union Canal?)	TQ2073	Richmond Park	TQ1583	Grand Union Canal, Perivale
TQ2966	Beddington Sewage Works	TQ3589	Walthamstow Reservoirs	TQ2867	Mitcham Common
TQ1684	Grand Union Canal	TQ0392	Maple Cross	TQ2983	Regent's Canal
TQ1268	Molesey Reservoirs	TQ3796	King George's Reservoir	TQ4087	Wanstead Park
TQ2783	Primrose Hill	TQ2966	Beddington Sewage Works	TQ2990	Alexandra Palace
TQ3589	Walthamstow Reservoirs	TQ2187	Brent Reservoir	TQ2668	Morden Hall Park
TQ2187	Brent Reservoir	TQ2472	Wimbledon Park	TQ3371	Crystal Palace

S3. The ten grid cells with the highest number of records of birds, of flowering plants, and of beetles.

Birds		Flowering plants		Beetles	
TQ2277	The London Wetland Centre	TQ2967	Mitcham Common	TQ3587	Walthamstow Marshes
TQ5280	Rainham Marshes Nature Reserve	TQ2868	Mitcham Common	TQ5379	Rainham Marshes RSPB Reserve
TQ2966	Beddington Farmlands	TQ2867	Mitcham Common	TQ5088	Romford
TQ1268	Molesley Reservoirs	TQ2972	Tooting Bec Common	TQ3799	Rammey Marsh/Gunpowder Park
TQ3589	Walthamstow Reservoir	TQ1974	Richmond Park	TQ3798	Enfield Lock
TQ2783	Regent’s Park	TQ2983	Regent’s Canal	TQ4379	Woolwich
TQ2187	Brent Reservoir	TQ3283	Regent’s Canal	TQ5282	Rainham Marshes Nature Reserve
TQ2780	Hyde Park	TQ2277	The London Wetland Centre	TQ5180	Rainham Marshes Nature Reserve
TQ0493	Stocker’s Lake	TQ3083	Camley Street Natural Park	TQ3579	Rainham Marshes Nature Reserve
TQ1669	Bushy Park	TQ5280	Rainham Marshes Nature Reserve	TQ4682	Creekmouth

S4. The ten most recorded species and their relative abundance within their taxonomic group in each data set for the informal taxonomic groups of birds, flowering plants and beetles.

Taxonomic group	Species from GiGL	Species from iSpot	Species from iRecord
Birds	Blackbird (2.15%)<br>*Turdus merula*	Grey heron (3.7%)<br>*Ardea cinerea*	Blackbird (8.74%)<br>*Turdus merula*
	Robin (2.06%)<br>*Erithacus rubecula*	Mallard (3.15%)<br>*Anas platyrhynchos*	Robin (7.66%)<br>*Erithacus rubecula*
	Tufted duck (1.83%)<br>*Aythya fuligula*	Robin (2.74%)<br>*Erithacus rubecula*	House sparrow (7.12%)<br>*Passer domesticus*
	House sparrow (1.77%)<br>*Passer domesticus*	Ring-necked parakeet (2.68%)<br>*Psittacula krameri*	Blue tit (6.05%)<br>*Cyanistes caeruleus*
	Sparrowhawk (1.65%)<br>*Accipiter nisus*	Tufted duck (2.62%)<br>*Aythya fuligula*	Woodpigeon (5.65%)<br>*Columba palumbus*
	Mallard (1.55%)<br>*Anas platyrhynchos*	Egyptian goose (2.56%)<br>*Alopochen aegyptiacus*	Magpie (4.17%)<br>*Pica pica*
	Woodpigeon (1.54%)<br>*Columba palumbus*	Black-headed gull (2.50%)<br>*Chroicocephalus ridibundus*	Ring-necked parakeet (3.76%)<br>*Psittacula krameri*
	Coot (1.51%)<br>*Fulica atra*	Moorhen (2.26%)<br>*Gallinula chloropus*	Swift (3.63)<br>*Apus apus*
	Wren (1.46%)<br>*Troglodytes troglodytes*	Coot (2.20%)<br>*Fulica atra*	Great tit (2.96%)<br>*Parus major*
	Chiffchaff (1.40%)<br>*Phylloscopus collybita*	Mute swan (2.20%)<br>*Cygnus olor*	Starling (2.96%)<br>*Sturnus vulgaris*
Flowering plants	Redshank (13%)<br>*Persicaria maculosa*	Herb-robert (1%)<br>*Geranium robertianum*	Japanese knotweed (5%)<br>*Fallopia japonica*
	Common ivy (5.5%)<br>*Hedera helix*	Pedunculate oak (0.8%)<br>*Quercus robur*	Common ivy (2.9%)<br>*Hedera helix*
	Common holly (1.6%)<br>*Ilex aquifolium*	Black horehound (0.7%)<br>*Ballota nigra*	Common nettle (2.9%)<br>*Urtica dioica*
	Pedunculate oak (1.2%)<br>*Quercus robur*	Spindle (0.7%)<br>*Euonymus europaeus*	Herb-robert (2.8%)<br>*Geranium robertianum*

	European ash (0.9%)<br>*Fraxinus excelsior*	Common mallow (0.7%)<br>*Malva sylvestris*	European ash (2.5%)<br>*Fraxinus excelsior*
	Sycamore (0.6%)<br>*Acer psuedoplatanus*	Green alkanet (0.7%)<br>*Pentaglottis sempervirens*	Ribwort plantain (2.3%)<br>*Plantago lanceolata*
	Common nettle (0.6%)<br>*Urtica dioica*	Common ragwort (0.7%)<br>*Jacobaea vulgaris*	Common hawthorn (2.2%)<br>*Crataegus monogyna*
	Common hawthorn (0.6%)<br>*Crataegus monogyna*	Bluebell (0.7%)<br>*Hyacinthoides non-scripta*	Daisy (2.2%)<br>*Bellis perennis*
	Common elder (0.5%)<br>*Sambucus nigra*	Lesser celandine (0.7%)<br>*Ranunculus ficaria*	Cleavers (2.1%)<br>*Galium aparine*
	Cow parsley (0.5%)<br>*Anthriscus sylvestris*<br>Cleavers (0.5%)<br>*Galium aparine*	Common birds-foot-trefoil (0.6%)<br>*Lotus corniculatus*<br>Red campion (0.6%)<br>*Silene dioica*	Red clover (1.9%)<br>*Trifolium pratense*
Beetles	Stag beetle (43%)<br>*Lucanus cervus*	Harlequin ladybird (17.5%)<br>*Harmonia axyridis*	Harlequin ladybird (52%)<br>*Harmonia axyridis*
	Seven spot ladybird (1.3%)<br>*Coccinella septempunctata*	Seven spot ladybird (4.8%)<br>*Coccinella septempunctata*	Seven spot ladybird (12%)<br>*Coccinella septempunctata*
	Twenty-two spot ladybird (1.0%)<br>*Psyllobora vigintiduopunctata*	Stag beetle (4.8%)<br>*Lucanus cervus*	Two spot ladybird (10%)<br>*Adalia bipunctata*
	Odemera lurida (0.9%)	*Oedemera nobilis* (4.7%)	Ten spot ladybird (3.8%)<br>*Adalia decempunctata*
	Sitona lineatus (0.9%)	Fourteen spot ladybird (3.0%)<br>*Propylea quattuordecimpunctata*	Twenty two spot ladybird (3.1%)<br>*Psyllobora vigintiduopunctata*
	Two spot ladybird (0.8%)<br>*Adalia bipunctata*	Rosemary leaf beetle (2.9%)<br>*Chrysolina americana*	Fourteen spot ladybird (2.6%)<br>*Propylea quattuordecimpunctata*
	Twenty-four spot ladybird (0.8%)<br>*Subcoccinella vigintiquattuorpunctata*	Lesser stag beetle (2.3%)<br>*Dorcus parallelipipedus*	Pine ladybird (2.6%)<br>*Exochomus quadripustulatus*
	Adonis ladybird (0.7%)<br>*Hippodamia variegata*	Varied carpet beetle (2.1%)<br>*Anthrenus verbasci*	Orange ladybird (2.1%)<br>*Halyzia sedecimguttata*
	Fourteen spot ladybird (0.7%)	Wasp beetle (2.1%)	Twenty-four spot ladybird (1.6%)

	Propylea quattuordecimpunctata	*Clytus arietis*	*Subcoccinella vigintiquattuorpunctata*
	Oedemera nobilis (0.6%)	Two spot ladybird (2.0%)<br>*Adalia bipunctata*<br>Rose chafer<br>*Cetonia aurata*<br>Spotted longhorn<br>*Rutpela maculata*	Eleven spot ladybird (1.2%)<br>*Coccinella undecimpunctata*
